# Urologists’ opinion on treating asymptomatic stones: Would we treat ourselves as we treat our patients? Survey from European Association of Urology, Young Academic Urologists, Endourology and Urolithiasis working party

**DOI:** 10.55730/1300-0144.5779

**Published:** 2023-12-07

**Authors:** Tarık Emre ŞENER, Thomas TAILLY, Yılören TANIDIR, Etienne Xavier KELLER, Amelia PIETROPAOLO, Juan Gomez RIVAS, Zeeshan HAMEED, Vincent DE CONINCK, Tzevat TEFİK, Kemal SARICA, Ali Serdar GÖZEN, Andreas SKOLARIKOS, Olivier TRAXER, Christian SEITZ, Bhaskar SOMANI

**Affiliations:** 1 Department of Urology, Marmara University, School of Medicine, İstanbul, Turkiye; 2 EAU, Young Academic Urologists (YAU), Endourology & Urolithiasis Working Group, Arnhem, The Netherlands; 3 Department of Urology, Ghent University Hospital, Ghent, Belgium; 4 EAU, Section of Uro-Technology (ESUT), Arnhem, The Netherlands; 5 Department of Urology, University Hospital Zurich, University of Zurich, Zurich, Switzerland; 6 Department of Urology, University Hospital Southampton NHS Foundation Trust, Southampton, United Kingdom; 7 Department of Urology, La Paz University Hospital, Madrid, Spain; 8 Department of Urology, Kasturba Medical College, Manipal Academy of Higher Education, Manipal, Karnataka, India; 9 Department of Urology, AZ Klina, Brasschaat, Belgium; 10 Department of Urology, Istanbul Faculty of Medicine, İstanbul University, İstanbul, Turkiye; 11 Department of Urology, Medicana Bahcelievler Hospital, Biruni University, İstanbul, Turkiye; 12 EAU, European Section of Urolithiasis (EULIS), Arnhem, The Netherlands; 13 Department of Urology, SLK-Kliniken Heilbronn, University of Heidelberg, Heilbronn, Germany; 14 European School of Urology (ESU), National and Kapodistrian University of Athens, Athens, Greece; 15 Department of Urology, National and Kapodistrian University of Athens, Athens, Greece; 16 Department of Urology, Tenon University Hospital, Sorbonne University, Paris, France; 17 Department of Urology, Comprehensive Cancer Center, Medical University of Vienna, Vienna, Austria

**Keywords:** Urolithiasis, survey, decision making, kidney, ureter

## Abstract

**Background/aim:**

Management of asymptomatic kidney stones is an ongoing debate with follow-up and treatment guidelines based on low-level evidence. Our aim was to evaluate current management of asymptomatic urinary stones.

**Materials and methods:**

A 70-question survey was designed in collaboration with European Association of Urology, Young Academic Urologists, Section of Uro-Technology and Section of Urolithiasis groups and distributed. Responders filled out hypothetical scenarios from 2 perspectives, either as treating physicians, or as patients themselves.

**Results:**

A total of 212 (40.01%) responses were obtained. Median responder age was 39 years. 75% of responders were interested in “urolithiasis”. 82.5% had never experienced a renal colic, 89.6% had never undergone urolithiasis treatment.

Overall, as the kidney stone scenarios got more complicated, the invasiveness of the treatment preference increased. As “the physician”, responders preferred the conservative option in all situations more than they would choose as “the patient”. For ureteral stones, conservative approach was most preferred for small stones and ureteroscopy became more preferred as the stone size increased.

For smaller kidney stones, the most preferred follow-up schedule was 4–6 monthly, whereas for larger and complicated stones it was 0–3 monthly from both perspectives respectively. For all ureteral stone scenarios, 0–4 weekly follow-up was mostly preferred.

Interestingly, having had a renal colic was an independent predictor of an interventional approach, whereas having had an intervention was an independent predictor of a conservative approach.

**Conclusion:**

Current treatment and follow-up patterns of asymptomatic urinary stones are in agreement with international guidelines on symptomatic stones.

In most of the urolithiasis situations urologists chose a conservative approach for their patients compared to what they would prefer for themselves. Conversely, urologists, in the scenarios as “the patient”, would like to have a more frequent follow-up schedule for their stones compared to how they would follow-up their patients.

## 1. Introduction

Along with the increased prevalence of urolithiasis, there is also an increase in the diagnosis of incidental asymptomatic stones, which can be linked to improvements in imaging technologies and the frequency of tests performed for check-up purposes [1–3]. In a series of 5047 asymptomatic patients screened for gastrointestinal purposes, incidental urinary stones were found at 7.8% [4].

As is apparent from the European Association of Urology (EAU) guidelines on urolithiasis, for nonobstructing asymptomatic renal stones, observation is a valid management option. However, the natural history of such stones is difficult to predict [5, 6].

While active treatment and active surveillance are both defendable management strategies, the approach should be the result of shared patient decision-making. [7]. Information about stone-free rates and complications of all possible treatment modalities, as well as the natural history of asymptomatic stones should be discussed. If a conservative approach is a valid option, short- and long-term risks, stone growth, and the ever-present possibility of evolving into acute situations should be considered. The idea behind conservative follow-up is to avoid unnecessary morbidity, while the argument for prophylactic intervention is to prevent the morbidity of an acute event, to have a better chance of achieving stone-free status when the patient is still fit for surgery [8].

As urinary stone disease is very prevalent around the world, every urologist will encounter stone patients, at least during residency, and has a basic knowledge of the possible treatment options and follow-up course [9, 10].

What to do with asymptomatic kidney stones remains a topic of ongoing debate that lacks high-level evidence. The goal of this survey was to evaluate the current management of asymptomatic urinary stones among urologists. Patients tend to rely on their physicians to educate them on alternative options and to actively participate in the decision-making process [11]. Therefore, we conducted a survey among urologists already trained with the necessary knowledge and experience about alternatives, success, and complications.

## 2. Materials & method

A 70-question online survey was designed by members of Young Academic Urologists (YAU) Endourology & Urolithiasis group after an initial consultation with EAU YAU board, EAU Section of Uro-Technology (ESUT) and EAU Section of Urolithiasis (EULIS) groups. The survey was designed and carried out in accordance with the Checklist for Reporting Results of Internet E-Surveys (CHERRIES) [12]. Before circulating, the survey was pilot tested for usability and technical functionality. Once approved, the survey was distributed via the Google Docs platform as an online survey. All the questions in the survey were labelled as “answer required to complete survey” in order to prevent any missing answers for any of the questions. Data was extracted from Google Docs platform and was transformed into an SPSS data sheet for SPSS Windows version 26.0 (IBM Corp., Armonk, NY, USA). The survey was distributed to YAU subgroup members, ESUT and EULIS board members, Progress in Endourology, Training and Research Association (PETRA) members, Progressive Endourological Association for Research and Leading Solutions (PEARLS) members, and faculty members of all the coauthors’ institutions. At the beginning of the survey, an explanatory text was provided and all the responders were asked not to fill out the survey in case they received it more than once from another source. The survey distribution commenced in January 2022, and the results were extracted from the database in July 2022, marking the conclusion of the study. The invitation was sent out only once, without sending a reminder.

The survey consisted of 5 subsections.

The 1^st^ section consisted of questions about demographic information, areas of interest in urological practice, and previous urolithiasis history of responders.

The 2^nd^ and 3^rd^ sections were about treatment choices in 14 situations with different stone locations and sizes in the kidney or ureter.

The 4^th^ and 5^th^ sections were about follow-up schedule choices in the same 14 situations with different stone locations and sizes.

According to stone location and sizes, 14 scenarios were provided (Table 1) along with the following presumptions as an ‘index patient’: “no symptoms; anatomically normal kidneys; normal kidney functions; no infectious signs and symptoms”.

In the 2^nd^ and 4^th^ sections the responders were asked to answer the questions considering themselves as the “treating physician”; and in the 3^rd^ and 5^th^ sections responders were asked to answer considering themselves as the “patient themselves” facing the same situations.

According to the invasiveness of the treatment options, the ranking was conservative, shock wave lithotripsy (SWL), ureteroscopy (URS), and percutaneous nephrolithotomy (PCNL), from the least to the most invasive option.

### 2.1. Statistical analysis

Sample distribution analysis was performed using a Shapiro-Wilk test and histograms. Categorical variables are expressed as counts and percentages, whereas continuous variables are expressed as medians, minimum, and maximum. McNemar-Bowker test was used to compare paired proportions with 3 or more categories. Logistic regression analyses were performed to identify demographic predictors of responders choosing either an interventional approach (SWL, URS, and PCNL), or a conservative approach. All statistical tests were two-sided with p values <0.05 considered statistically significant. Participants’ responses to different clinical scenarios were shown on stacked bar graphs as the “patient” and the “physician” to emphasize the difference in treatment choice and follow-up schedules. Intra-rater agreement between the “patient” and the “physician” situations for each scenario was expressed as a percentage and calculated by recoding the number of responses where the responder chose the same answer in both situations as the “patient” and the “physician” for all the possible answer choices. Analyses were performed with SPSS for Windows version 26.0 (IBM Corp., Armonk, NY, USA).

## 3. Results

The survey was distributed to a total number of 528 urologists. A total of 212 responses were obtained, giving a responder rate of 40.01% and all the data was analysed accordingly. In 212 response received, none of the questions had a missing answer because all the questions were labelled as “answer required to complete survey”. Median responder age was 39 years. Demographic information is given in Table 2. 159 (75%) responders had main interest in “urolithiasis”. 175 (82.5%) had never experienced a renal colic and 89.6% had never undergone any kind of urolithiasis treatment. The frequency of the stone treatment options performed per year is categorised in Table 3.

**Figure 1:**
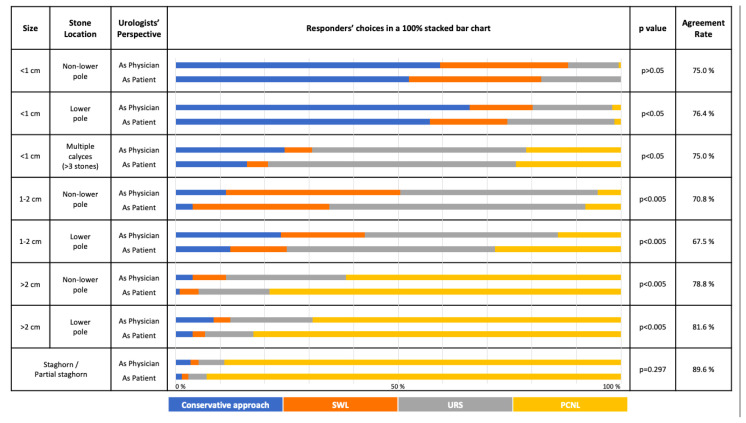
The choice of different treatment approaches in different asymptomatic kidney stone scenarios considering the responder is either “the treating physician” or “the patient” him/herself (SWL: Shockwave Lithotripsy; URS: Semirigid/Flexible Ureteroscopy; PCNL: Mini/standard

**Figure 2:**
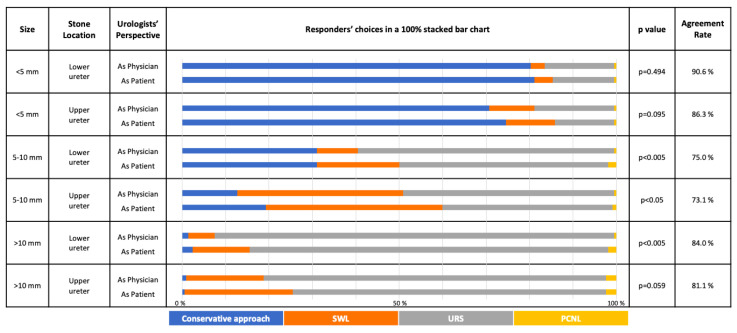
The choice of different treatment approaches in different asymptomatic ureteral stone scenarios considering the responder is either “the treating physician” or “the patient” him/herself (SWL: Shockwave Lithotripsy; URS: Semirigid/Flexible Ureteroscopy; PCNL: Mini/standard Percutaneous Nephrolithotomy; Conservative Approach: Follow-up with/without Medical Expulsive Therapy).

**Figure 3:**
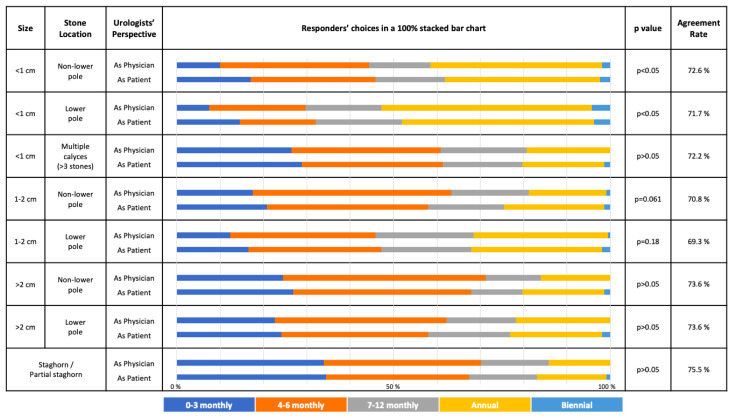
The choice of different follow-up schedules in different asymptomatic kidney stone scenarios considering the responder is either “the treating physician” or “the patient” him/herself (Annual: Once every year; biennial: Once every 2 years).

**Figure 4:**
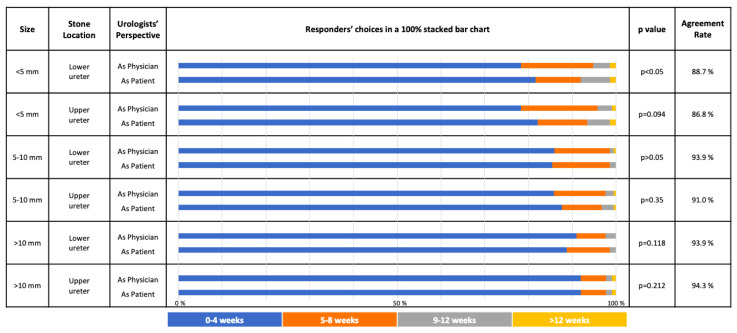
The choice of different follow-up schedules in different asymptomatic ureteral stone scenarios considering the responder is either “the treating physician” or “the patient” him/herself (Annual: Once every year; biennial: Once every 2 years).

The overall choices of responders for both treatment and follow-up schedule options in both kidney and ureteral stone scenarios are given in Figures 1, 2, 3, and 4 respectively.

### 3.1. According to size and location

In kidney stone scenarios, as the size of the stone increased, the invasiveness of the preferred treatment option also increased. For solitary stones <1 cm, most of the responders (>50%) opted for a conservative approach from both perspectives. However, for multiple stones <1 cm, the most employed option was URS. For kidney stones 1–2 cm, the most preferred option was URS, whereas in the nonlower pole stones, SWL was also given as an option. Most of the responders (>50%) preferred PCNL for stones >2 cm and for staghorn/partial staghorn stones (Figure 1).

For both lower and nonlower pole stones <1 cm, most of the responders preferred an annual follow-up schedule both “as physician” and “as patient”. For every situation with stone >1 cm in both lower and nonlower pole, the most common follow-up schedule option was 4–6 monthly from both perspectives (Figure 3).

In ureteral stone scenarios, most of the responders (>50%) chose conservative approach for stones <5 mm while considering themselves as both “the physician” and “the patient”. In distal ureteral stones 5–10 mm, URS and conservative approach were the most preferred options; and in proximal ureteral stones 5–10 mm, URS and SWL were mostly preferred (Figure 2).

Majority of the responders (>75%) chose a close follow-up schedule for both their patients and for themselves as the patient, with 0–4 weekly follow-up being the most common preferred option in both proximal and distal ureteral stones (Figure 4).

### 3.2. Overall distribution

Most of the preferred treatment selections in different clinical scenarios had statistically significant differences between “the physician” and “the patient” perspectives (Figures 1 and 2).

No evidence of a significant difference (p > 0.05) in treatment options was found for the following situations: Nonlower pole kidney stone <1 cm, Staghorn/partial staghorn stone, distal ureteral <5 mm, proximal ureteral stone <5 mm and >10 mm.

For most of the follow-up schedule options, the choices did not have a significant difference in both perspectives (Figures 3 and 4).

In the following situations, the responders’ choices were significantly different (p < 0.05): Nonlower pole kidney stone <1 cm, Lower pole kidney stone <1 cm, distal ureteral <5 mm.

When data was analysed for intra-rater agreement rates:

For the treatment options of kidney and ureteral stones, the highest agreement rates between the 2 groups were found for “Staghorn/partial staghorn stones” (89.6%) and “Lower ureteral stone <5 mm” (90.6%), respectively. Conversely, the highest disagreement for kidney and ureteral stones were found for “Lower pole stone 1–2 cm” (67.5%) and “Upper ureteral stone 5–10 mm” (73.1%), respectively.

For the follow-up schedule options of kidney and ureteral stones, the highest agreement rates between 2 groups were found for “Staghorn/partial staghorn stones” (75.5%) and “Upper ureteral stone >10 mm” (94.3%), respectively. Conversely, the highest disagreement for kidney and ureteral stones were found for “Lower pole stone 1–2 cm” (69.3%) and “Upper ureteral stone <5 mm” (86.8%), respectively.

Binary logistic regression tests revealed that, of all demographic parameters from the responders, having had a renal colic was an independent predictor of choosing an interventional approach (OR 1.5, 95% CI 1.1–2.0; p=0.01) in both “the physician” and “the patient” scenarios, whereas having had an intervention was an independent predictor of preferring a conservative approach (OR 1.01, 95% CI 1.01–1.19; p = 0.04) in both scenarios. All other demographic parameters were not predictive.

## 4. Discussion

According to EAU guidelines on urolithiasis, there are different approaches to kidney stones depending on size, location, and patient characteristics [6]. In any situation where multiple management options can be confidently offered, a shared decision-making process should be undertaken with a well-informed patient.

Asymptomatic renal stones have variable rates of progression to a symptomatic event (28%–77%) and variable rates of need for intervention (7.1%–26%) [13–16]. Most of the studies evaluating the success rates of different treatment modalities on asymptomatic stones are based on lower pole stones [17]. For 1–2 cm lower pole stones, EAU guidelines have a separate treatment algorithm lacking sharp outlines [6]. This was also reflected in our study, where the decisions on 1–2 cm lower pole stones as “the physician” and as “the patient” had significant differences with URS being the most commonly selected option. For large stones (>2 cm), in any calyx, the treatment approach was in concordance with EAU guidelines and the responders’ first choice was PCNL. However, responders preferred a more minimally invasive approach (URS) for their patients where they mostly chose PCNL for themselves as “the patient”.

The guidelines recommend active removal of kidney stones in cases of growth, in high-risk stone formers, obstruction, infection, symptoms (e.g., pain or hematuria), stones of >15 mm, stones of <15 mm if observation is not an option, patient preference, comorbidity, social situation (e.g., profession or travelling) [6]. For stones <1 cm inside the kidney, the most preferred option was the conservative approach followed by SWL. SWL was also one of the highly recommended treatment options according to EAU guidelines [6]. For ureteral stones, the significant differences among choices as “the patient” and “the physician” were observed for stones 5–10 mm. Both EAU and AUA guidelines have a threshold of 10 mm for categorization of ureteral stones and do not have <5 mm and 5–10 mm subcategories. For ureteral stones >10 mm, the recommendation is URS. In our survey, the most common preferred option for stones <5 mm was the conservative approach without any significant differences between the 2 perspectives. For ureteral stones 5–10 mm, the most preferred option was URS however there was a significant difference between “as physician” and “as patient” perspectives and the responders preferred to perform URS for their patients, more than they would undergo URS themselves. In larger ureteral stones (>10 mm), the preferred choices were in concordance with the guidelines and the majority of the responders chose URS in both perspectives without any significant differences.

In all kidney stone scenarios with >1 cm stones and in all ureteral stone scenarios with stones >5 mm, the responders chose to undergo at least one of the treatment options rather than a conservative approach. The reason for choosing an invasive option may be technological advancements that provide successful outcomes with high stone-free rates and low complication rates [18, 19]. Apart from the potential acute situation, this successful treatment option is one of the reasons that patients and urologists would want to get rid of a stone even though it is asymptomatic.

In a previous survey, it was reported that urolithiasis patients tend to prefer their physician to actively play a role in selecting the appropriate treatment for their asymptomatic stones. The authors have also reported that previous stone experiences had an influence on treatment selection and that the patients were more likely to choose what they were already familiar with [11]. Similarly, we also found out that previous renal colic experience was an independent predictor of choosing an interventional approach, and having undergone an intervention was an independent predictor of preferring a conservative approach.

AUA and EAU guidelines state that surveillance is an option in asymptomatic nonobstructing stones, but currently give no recommendations on the optimal modality or frequency of surveillance imaging [5, 6]. In our study, the follow-up schedule options for “the physician” and “the patient” were mostly similar without any statistically significant differences. The only significant differences were observed with <1 cm kidney stones (both lower and nonlower poles) and <5 mm lower ureteral stones. For <1 cm kidney stones, they would prefer to be closely followed-up as “the patient” and chose a 0–3 monthly follow-up compared to the scenario where they were “the physician”.

It has been previously demonstrated that the gold standard treatment for staghorn stones is PCNL [20, 21]. In our study, we also can see that “Staghorn/partial staghorn stones” have the highest agreement rates between the 2 groups for both treatment and follow-up schedule options. Conversely, in literature, the “Lower pole stones of size 1–2 cm” treatment and follow-up is a topic of debate [22], and both treatment and follow-up schedule options for “Lower pole stone 1–2 cm” had the highest disagreement rates in our survey. Whereas the responders would lean towards active treatment rather than a conservative approach from the patient’s perspective for the lower pole stone scenario of 1–2 cm, the opposite holds true for the upper ureteral stone of 5–10 mm. In that scenario, the responders would act more conservatively from a patient’s perspective.

In modern medicine, apart from treating the disease, it is also important to improve the quality of life (QoL) of our patients. Therefore, treatment choices in different clinical scenarios may also vary according to previous urolithiasis experience of the responders.

### 4.1. Limitations of the study

The survey was not a validated questionnaire. However, it was designed by Young Academic Urologists (YAU) Endourology & Urolithiasis group in conjunction with ESUT and EULIS groups and approved by the relevant authorities of these groups, so validation was not considered a required process.

## 5. Conclusion

Current treatment and follow-up patterns of asymptomatic urinary stones are in concordance with international guidelines on symptomatic stones. Interestingly, in most urolithiasis situations, whether in kidney or ureter, the urologists tend to offer a slightly more conservative approach for their patients as their “physician” compared to what they would choose for themselves as “the patient”. Conversely, urologists, in the scenarios as “the patient”, would like to have a more frequent follow-up schedule for their urinary stones compared to how they would follow-up their urolithiasis patients.

## Figures and Tables

**Table 1:** Structural configuration of survey scenarios.

Location		Cumulative stone size in diameter
Kidney	Solitary nonlower pole Solitary lower pole Multiple stones in different calyces Staghorn/partial staghorn	<1 cm 1–2 cm >2 cm
Ureter	Lower ureter Upper ureter	<5 mm 5–10 mm >10 mm
Age (median, min, max)	39 (24, 84)	
Years of practice in urology (%)	<10 years	42%
	10–20 years	32.1%
	20–30 years	15.1%
	>30 years	10.8%
Institution (%)	University hospital	70.8%
	Public hospital	12.2%
	Private hospital/clinic	17%

**Table 2:** Demographic information on responders (n: 212).

Age (median, min, max)	39 (24, 84)	
Years of practice in urology (%)	<10 years	42%
	10–20 years	32.1%
	20–30 years	15.1%
	>30 years	10.8%
Institution (%)	University hospital	70.8%
	Public hospital	12.2%
	Private hospital/clinic	17%
Interest in “Urolithiasis” treatment (%)	Yes	75%
	No	25%
Patients’ insurance status in country of residence (%)	<25% of patients are insured	9.9%
	26%–50% of patients are insured	5.2%
	51%–75% of patients are insured	10.8%
	>75% of patients are insured	74.1%

**Table 3:** The frequency of performing a specific urolithiasis treatment option.

Shockwave lithotripsy	<25 cases / year	51.9%
	25–100 cases/year	26.6%
	>100 cases/year	21.2%
Ureteroscopy	<25 cases/year	6.6%
	25–100 cases/year	37.7%
	>100 cases/year	55.7%
Percutaneous nephrolithotomy	<25 cases/year	27.4%
	25–100 cases/year	50.9%
	>100 cases/year	21.7%
